# TempShift Reveals the Sequential Development of Human Neocortex and Skewed Developmental Timing of Down Syndrome Brains

**DOI:** 10.3390/brainsci13071070

**Published:** 2023-07-13

**Authors:** Yuqiu Zhou, Li Tao, Ying Zhu

**Affiliations:** State Key Laboratory of Medical Neurobiology, MOE Frontiers Center for Brain Science, Institutes of Brain Science and Department of Neurosurgery, Huashan Hospital, Fudan University, Shanghai 200032, China; 20211520004@fudan.edu.cn (Y.Z.); 22211520042@m.fudan.edu.cn (L.T.)

**Keywords:** cortical development, synaptogenesis, Down syndrome, temporal dynamics

## Abstract

Development is a complex process involving precise regulation. Developmental regulation may vary in tissues and individuals, and is often altered in disorders. Currently, the regulation of developmental timing across neocortical areas and developmental changes in Down syndrome (DS) brains remain unclear. The changes in regulation are often accompanied by changes in the gene expression trajectories, which can be divided into two scenarios: (1) changes of gene expression trajectory shape that reflect changes in cell type composition or altered molecular machinery; (2) temporal shift of gene expression trajectories that indicate different regulation of developmental timing. Therefore, we developed an R package TempShift to separates these two scenarios and demonstrated that TempShift can distinguish temporal shift from different shape (DiffShape) of expression trajectories, and can accurately estimate the time difference between multiple trajectories. We applied TempShift to identify sequential gene expression across 11 neocortical areas, which suggested sequential occurrence of synapse formation and axon guidance, as well as reconstructed interneuron migration pathways within neocortex. Comparison between healthy and DS brains revealed increased microglia, shortened neuronal migration process, and delayed synaptogenesis and myelination in DS. These applications also demonstrate the potential of TempShift in understanding gene expression temporal dynamics during different biological processes.

## 1. Introduction

Gene expression has been observed to be dynamically regulated across development in many organisms. The temporal dynamics are often associated with the occurrence of developmental processes, such as generating new cells, responding to internal or external signals, and so on. While some of the developmental processes are universal, some others are distinct to species, tissues or disorders, which are accompanied by different temporal gene expression patterns. The differences during development can be categorized into two scenarios: (1) different shape (DiffShape) of gene expression trajectories may reflect increase or decrease in certain cell types, and disrupted or altered molecular machinery of developmental processes; (2) temporal shift of gene expression trajectories reflect different regulation of developmental timing.

Development of the cerebral neocortex is an example. The neocortex is organized into structurally similar but functionally distinct areas. Development of neocortical areas involves in common processes, such as neurogenesis, neuron differentiation, synaptogenesis, myelination, and so on, but the maturation rate of distinct neocortical areas has been found different. Furthermore, these neurodevelopmental disorders have been observed impaired in neurodevelopmental disorders, such as Down syndrome (DS). DS, also known as trisomy 21, is a genetic disorder caused by the presence of all or part of a third copy of chromosome 21. As the most common neurodevelopmental disorder, the estimated prevalence of DS is as high as 13.65 per 10,000 live births [1]. DS is typically characterized by delays of physical growth and intellectual disabilities. Morphometric and cellular studies in human and functional studies in mouse models have indicated defects in neurogenesis, neuronal differentiation, synaptic plasticity and myelination [2,3,4]. However, understanding the regulation of developmental timing across neocortical areas and defects in DS remains challenging.

**Figure 1:**
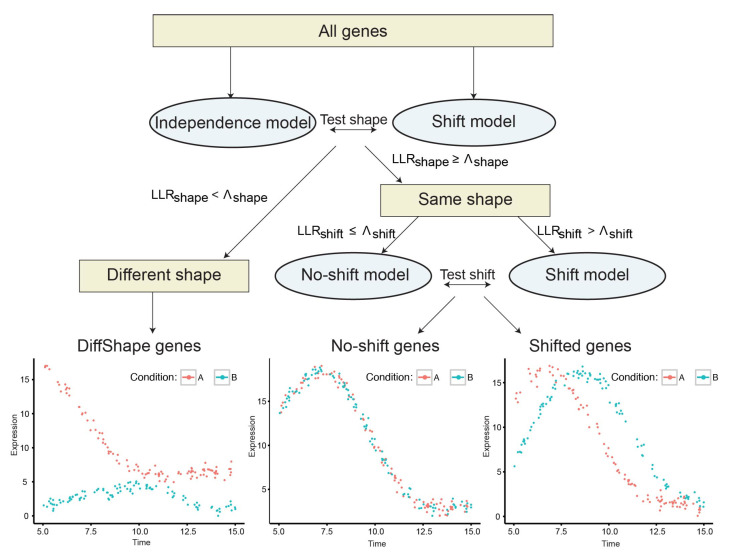
Workflow of TempShift. Different colors (red and blue) indicate the time-series data of gene expression under different conditions.

The time-series design of transcriptome analyses provides a unique opportunity to detect the two scenarios of developmental changes in a high-throughput way. To date, multiple approaches have been developed for differential expression analysis, clustering and alignment of time-series data [5]. A Gaussian process-based model, TempShift (Appendix A) has been proposed to distinguish the above two scenarios in our previous study on spatiotemporal transcriptomic divergence across human and macaque brain development [6]. TempShift divided the genes into three categories: DiffShape genes—these genes follow different shapes of expression trajectories under different conditions, shift genes—these genes express at different times without changing the expression trajectories over time under different conditions, and no-shift genes—these genes express at the same time and follow the same expression trajectories under different conditions (Figure 1). TempShift used Gaussian process [7] to model the gene expression trajectories so that it has the following advantages [8,9]: (1) it explicitly addresses the dependencies between consecutive measurements and thus can deal with non-matched time-point sampling in different groups; (2) it can handle an arbitrary number of replicates; (3) it provides a statistical framework to distinguish DiffShape genes from shift genes and no-shift genes, and to infer the time shift between groups; (4) it can be used to analyze two or more groups.

Here, we programmed TempShift into an R package. The performance of TempShift was tested using different types of simulated data first. Then, we applied TempShift to human brain transcriptome data including 11 neocortical areas, revealing the gradual development of the human neocortex. Further application to a DS dataset demonstrated the ability of TempShift in handling data with unmatched age and revealed increased microglia and skewed developmental timing of neuronal and oligodendrocyte development in DS.

## 2. Materials and Methods

### 2.1. TempShift: A Statistical Model to Detect Global Temporal Shift between Multiple Time Series

TempShift is a statistical model designed for two goals: (1) distinguishing DiffShape, shift and no-shift genes, and (2) for shift genes, estimating the time shift between groups. To achieve the above goals, it builds three temporal expression models based on Gaussian process regression: independence model, shift model and no-shift model (Figure 1; Appendix A). The DiffShape genes are first separated from genes with the same shape of trajectories (shift and no-shift genes) by comparing the independence model with the shift model, and the shift genes are then selected by comparing the shift model with the no-shift model. These comparisons are based on the log likelihood ratio of the shift model versus the independence model (${\mathrm{LLR}}_{shape}$) and the log likelihood ratio of the shift model versus the no-shift model (${\mathrm{LLR}}_{shift}$), respectively (Appendix A). Genes with ${\mathrm{LLR}}_{shape}$ smaller than a threshold $\Lambda_{shape}$ are selected as DiffShape genes; genes with ${\mathrm{LLR}}_{shape}$ greater than $\Lambda_{shape}$ and ${\mathrm{LLR}}_{shift}$ greater and $\Lambda_{shift}$ are selected as shift genes, and the rest genes are no-shift genes. The time shift (Δt) with respect to the reference group is estimated in the shift model.

### 2.2. Simulation

#### 2.2.1. Gaussian Process

For group *i*, the time vectors $x_{i}$ consist of 100 points randomly sampled from the uniform distribution between 5 and 15. For each model, we generated 100 genes with rmvnorm function in the mvtnorm R package [10], using their corresponding covariance matrix. The length of each gene expression vector is 100, corresponding to 100 time points of expression. The following parameters were used in the Gaussian kernel: the amplitude $\sigma_{f}=5$ and the length scale l = 3. The $\sigma_{f}$ determines the average distance of the function away from its mean. For two-group data, Δt between two groups were fixed to 2. For three-group data, the first group was considered as the reference group, and the time shifts of the other two groups with respect to the reference group were randomly sampled from Gaussian distribution N(2,1) or N(−2,1).

#### 2.2.2. Periodic Data

The sine function sin(·) is a well-known periodic function, so we can simulate periodic data based on the sine function. For group *i*, the time vectors $x_{i}$ consists of 100 points randomly sampled from the uniform distribution between 5 and 15. For both the no-shift model and the shift model, we simulated 100 genes each. The length of each gene expression vector is 100, corresponding to 100 time points of expression. Two-group and three-group data were simulated. For the no-shift model, the gene expression of each group was modeled as $y_{i}=\sin\left(\frac{2\pi x_{i}}{10}\right)+\epsilon_{i}$, with each element of the error vector $\epsilon_{i}$ following independent and identically distributed N(0,0.3). For the shift model, the first group (*i* = 1) was considered as the reference group, and was modeled by the same function as the no-shift model. For group i(i≠1), the gene expression was modeled as $y_{i}=\sin\left(\frac{2\pi (x_{i}-\Delta t_{i}\vec{1_{n_{i}}})}{10}\right)+\epsilon_{i}$. $\Delta t_{i}$ is the time shift between group *i* and the reference group; $\vec{1_{n_{i}}}$ is a vector of all ones with length of $n_{i}$, the sample size of group *i*; $\epsilon_{i}$ is the error vector with all elements following independent and identically distributed Gaussian distribution N(0,0.3).

#### 2.2.3. Polynomial Data

For each group, the time vectors $x_{i}$ were generated in the same way as in the Gaussian process and periodic data. Two-group and three-group data were simulated. For the no-shift model, gene expression was modeled as $y_{i}=a+\;b\times \left(x_{i}-10\right)+\;c\times {\left(x_{i}-10\right)}^{2}+\varepsilon_{i}$, for group *i*. The elements of $\epsilon_{i}$ follow independent and identically distributed N(0,3). For the shift model, gene expression of the reference group was simulated by the same formula as in the no-shift model, and gene expression of other groups (i≠1) was generated by $y_{i}=a+\;b\times \left((x_{i}-\Delta t_{i}\vec{1_{n_{i}}})-10\right)+\;c\times {\left((x_{i}-\Delta t_{i}\vec{1_{n_{i}}})-10\right)}^{2}+\varepsilon_{i}$, where $\Delta t_{i}$ is the time shift between group *i* and the reference group, and $\epsilon_{i}$ is the error vector with all elements following independent and identically distributed Gaussian distribution N(0,3).

### 2.3. Identifying Developmental Sequences in the Human Neocortex

Normalized human brain microarray data were downloaded from GSE25219 [11]. Samples from 11 neocortical areas were used for analysis. The samples used in the analysis were listed in Supplementary Table S1. Genes with temporal dynamics were pre-selected by cubic regression *gene expression* ∼$1+age+age^{2}+age^{3}$, where $age=\log_{2}\left(\mathrm{postconceptual}\;\mathrm{days}\right)$. A total of 4153 genes with variance explained by the cubic model $R^{2}>\;0.5$ were selected for further analysis by TempShift. The $R^{2}$ of cubic model measures the relationship between gene expression and developmental time. The larger the $R^{2}$, the greater the change in gene expression with developmental time. Therefore, we selected the genes with $R^{2}>\;0.5$ as input temporal dynamics genes to the downstream analysis with TempShift. Next, temporally shifted genes were selected with LLR threshold $\Lambda_{shape}>50$ and $\Lambda_{shift}>50$. Enrichment analysis was performed by PANTHER Overrepresentation Test (release 20160321) (http://pantherdb.org/, accessed on 18 April 2016) [12].

### 2.4. Identifying Developmental Changes in the down Syndrome

#### 2.4.1. Gene Selection

Microarray data of DS samples were downloaded from GSE59630 [13]. DFC samples were used and combined with the human DFC samples from GSE25219 in the above section. No obvious batch effect was observed between two data sets, as the same platform and normalization methods were applied. All 17,542 genes were analyzed by TempShift. DiffShape genes were selected with ${\mathrm{LLR}}_{shape}$ below two standard deviations below the mean and shift genes were selected with ${\mathrm{LLR}}_{shape}$ above mean and ${\mathrm{LLR}}_{shift}$ above 10.

#### 2.4.2. Cell Type Enrichment

Single cell RNA-seq of human neocortex were downloaded from GSE67835 [14]. Log2-RPM (reads per million mapped reads) were used to measure expression. To assign cell types, we first reduced the dimension of the data by tSNE and then performed k-means clustering. The cell type of a cluster of cells is determined by the expression of cell-type markers. We identified two clusters of prenatal single cells, representing progenitors and neurons, respectively, and six clusters of adult single cells, including pyramidal neuron, interneurons (two clusters), oligodendrocytes, astrocytes and microglia. For each gene, cell type enrichment was calculated by one-way ANOVA followed by post hoc Tukey’s honest significant difference (HSD) test. A gene is enriched in cell type A, if ANOVA *p* < 0.01 and Tukey’s HSD test *p* < 0.01 for comparison between cell type A and at least 6 other cell types. The enrichment of cell type-enriched in DiffShape genes and shift genes was tested by Fisher’s exact test.

## 3. Results

### 3.1. Testing TempShift by Simulation

To test the performance of TempShift [6] (https://github.com/YingZhuLab/TempShift, accessed on 31 May 2023) implemented in R, we applied it to different types of simulated data with known time shift, including Gaussian process data, periodic data, and polynomial data. The specific principles of data simulation are described in Section 2.

#### 3.1.1. Gaussian Process Data

**Figure 2:**
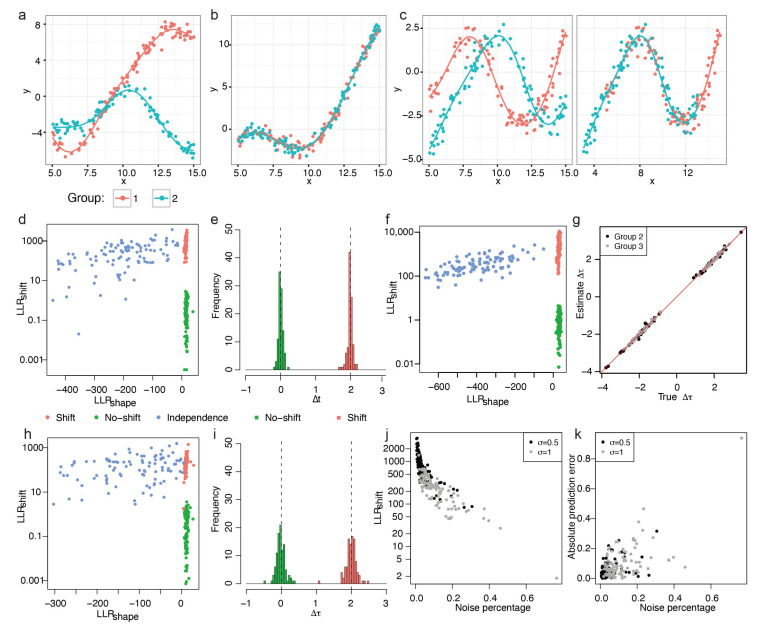
Application of TempShift to data simulated from Gaussian process. (**a**) An example of genes following the independence model in the two-group simulated data. The solid lines show the true trajectories. (**b**) An example of simulated genes following the no-shift model in the two-group simulated data. (**c**) An example of simulated genes following the shift model in the two-group simulated data (Left: before shifting; right: after shifting). (**d**,**e**) Simulated results of two-group data. σ=0.5. (**d**) The ${\mathrm{LLR}}_{shape}$ and ${\mathrm{LLR}}_{shift}$ of all two-group simulated genes. The ${\mathrm{LLR}}_{shift}$ is in logarithmic scale. The genes following the independence model are in blue; genes following the no-shift model are in green and genes following the shift model are in red. (**e**) The histogram of estimated Δt. The no-shift model is in green and the shift model is in red. The dashed lines show the true Δt of the no-shift (Δt=0) and the shift model (Δt=2). (**f**,**g**) Simulated results of three-group data. σ=0.5. (**f**) The ${\mathrm{LLR}}_{shape}$ and ${\mathrm{LLR}}_{shift}$ of all three-group simulated genes. The ${\mathrm{LLR}}_{shift}$ is in logarithmic scale. (**g**) The true Δt vs. the estimated Δt of group 2 (black) and group 3 (grey) compared with the reference group (group 1). The red line represents the line of estimated Δt = true Δt. (**h**,**i**) Simulated results of two-group data. σ=1. (**h**) ${\mathrm{LLR}}_{shape}$ and ${\mathrm{LLR}}_{shift}$ of all two-group simulated genes with high noise. The ${\mathrm{LLR}}_{shift}$ is plotted in logarithmic scale. (**i**) The histogram of estimated Δt. The no-shift model is in green and the shift model is in red. The dashed lines show the true Δt of the no-shift (Δt=0) and the shift model (Δt=2). (**j**) ${\mathrm{LLR}}_{shift}$ reduces with the increase in noise. (**k**) The prediction error increases with the increase in noise.

We first tested the performance of our model on two-group and three-group simulated data generated from Gaussian process. We generated 100 genes following the independence model (Figure 2a, Equation (A1)), the no-shift model (Figure 2b, Equation (A2)), and the shift model (Figure 2c, Equation (A3)), respectively (Appendix A), with noise term σ=0.5. The *x*-axis is the simulation time and the *y*-axis is the simulated gene expression.

In both the two-group and the three-group data, we observed clear separation between genes following the independence model and genes following the no-shift or shift model by ${\mathrm{LLR}}_{shape}$ and further separation between shift genes and no-shift gene by ${\mathrm{LLR}}_{shift}$ (Figure 2d,f). Therefore, based on ${\mathrm{LLR}}_{shape}$ and ${\mathrm{LLR}}_{shift}$, we can achieve the purpose of screening out DiffShape, no-shift and shift genes. Furthermore, our model accurately predicted the time shift in the shift model, with small mean squared prediction error (MSPE; two-group MSPE = 0.0061; three group MSPE = 0.0039 for group 2 and MSPE = 0.0027 for group 3; Figure 2e,g). The mean squared prediction error measures the expected squared distance between our estimated time shift and the true time shift.

Next, we analyze the robustness of the TempShift to noise. Residual variance refers to the variance in a model that cannot be explained by the variables in the model. The higher the residual variance of a model, the higher the noise level. In the above two-group data with σ=0.5, the residual variance constituted 0.3–65.2% total variance in the no-shift model (6.4 ± 9.8%) and 0.4–30.5% (5.7 ± 5.5%) in the shift model, where TempShift successfully distinguished all shift genes from no-shift genes under this noise level. To further explore the effects of noise level, we then increased the residual variance by setting σ=1, which resulted in the residual variance constituting 2.1–67.2% total variance in the no-shift model (16.7±14.8%) and 1.7–76.4% (11.9 ± 10.8%) in the shift model. In this case, TempShift is also able to distinguish most temporally-shifted genes from no-shift genes, except one gene with 76.4% noise (Figure 2h,i). In general, ${\mathrm{LLR}}_{shift}$ decreases and the prediction error increases with the level of noise (Figure 2j,k), and TempShift is robust to noise.

#### 3.1.2. Periodic Data

**Figure 3:**
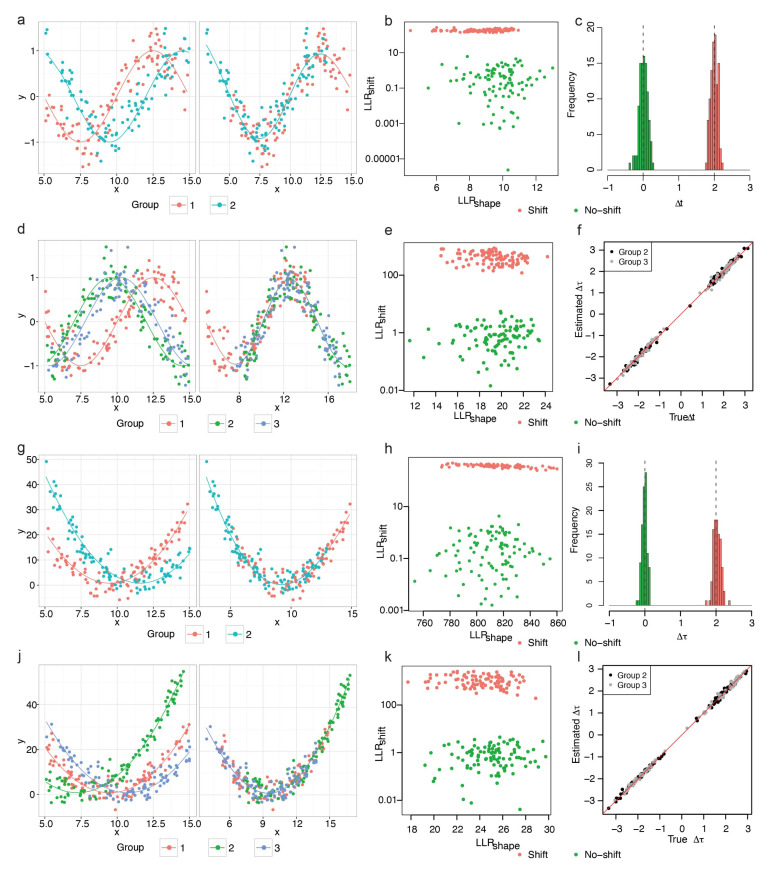
Application of TempShift to periodic and quadratic data. (**a**–**f**) Data simulated from sine function. (**a**–**c**) Two-group model. (**a**) An example of two-group shift gene. The solid lines show the trajectories of the sine function that generate the data. Left: before shifting and right: after shifting. (**b**) The ${\mathrm{LLR}}_{shape}$ and ${\mathrm{LLR}}_{shift}$ of all two-group simulated periodic genes. The ${\mathrm{LLR}}_{shift}$ was plotted in logarithmic scale. The shift genes are in green and the no-shift genes are in red. (**c**) The histogram of estimated Δt. The no-shift model is in green and the shift model is in red. The dashed lines showed the true Δt of the no-shift (Δt=0) and the shift model (Δt=2). (**d**–**f**) Three-group model. (**d**) An example of three-group shift genes. The solid lines show the trajectories of the sine function that generate the data. Left: before shifting and right: after shifting. (**e**) The ${\mathrm{LLR}}_{shape}$ and ${\mathrm{LLR}}_{shift}$ of all three-group simulated periodic genes. The ${\mathrm{LLR}}_{shift}$ is plotted in logarithmic scale. The no-shift genes are in green and the shift genes are in red. (**f**) The true Δt vs. the estimated Δt of group 2 (black) and group 3 (grey) compared with the reference group (group 1). The red line represents the line of estimated Δt = true Δt. (**g**–**l**) Data simulated from quadratic function. (**g**–**i**) Two-group model. (**g**) A representative two-group shift gene simulated from quadratic model. The solid lines show the trajectories of the quadratic function that generate the data. Left: before shifting and right: after shifting. (**g**) The ${\mathrm{LLR}}_{shape}$ and ${\mathrm{LLR}}_{shift}$ of all two-group simulated non-periodic genes. The ${\mathrm{LLR}}_{shift}$ is plotted in logarithmic scale. The no-shift genes are in green and the shift genes are in red. (**i**) The histogram of estimated Δt. The no-shift model is in green and the shift model is in red. The dashed lines showed the true Δt of the no-shift (Δt=0) and the shift model (Δt=2). (**j**–**l**) Three-group model. (**j**) A representative three-group shift gene. The solid lines show the trajectories of the quadratic function that generate the data. Left: before shifting and right: after shifting. (**k**) The ${\mathrm{LLR}}_{shape}$ and ${\mathrm{LLR}}_{shift}$ of all three-group simulated non-periodic genes. The ${\mathrm{LLR}}_{shift}$ is plotted in logarithmic scale. The no-shift genes are in green and the shift genes are in red. (**l**) The true Δt vs. the estimated Δt of group 2 (black) and group 3 (grey) compared with the reference group (group 1). The red line represented the line of estimated Δt = true Δt.

We then assessed whether TempShift could be applied to data generated from other models. TempShift was first applied to simulated periodic data, the expression changes associated with periodic processes, such as cell cycle or circadian rhythm. The residual variance constituted approximately 15% of the total variance in the simulated datasets. This percentage of noise is selected according to previous estimation in the microarray experiments [15]. For two-group data, we generated 100 no-shift genes (Δt=0) and 100 shift genes (Δt=2; Figure 3a), and for three-group data, we generated 100 no-shift genes (Δt=0) and 100 shift genes with the randomly sampled time shift (Δt) (Appendix A; Figure 2d). In both settings, TempShift successfully distinguished the shift genes from no-shift genes by ${\mathrm{LLR}}_{shift}$, and accurately estimated Δt (two-group data MSPE = 0.0095; three-group data MSPE = 0.0085 for $\Delta t_{1}$ and 0.0097 for $\Delta t_{2}$) (Figure 3b,c,e,f).

#### 3.1.3. Polynomial Data

The non-periodic data representing gene expression changes across development were simulated with a residual variance accounting for approximately 10%. Then, TempShift was applied to the simulated non-periodic data with two-group and three-group, respectively. Again, TempShift successfully distinguished the shift genes from no-shift genes with ${\mathrm{LLR}}_{shift}$ and accurately estimated Δt using both two-group (MSPE = 0.012) and three-group data sets (MSPE = 0.0052 for $\Delta t_{1}$ and MSPE = 0.0065 for $\Delta t_{2}$) (Figure 3h,i,k,l).

In summary, the simulation results demonstrated that TempShift is able to identify temporally shifted genes and accurately estimate time shift between groups even with high noise.

### 3.2. Identifying Developmental Sequences in the Human Neocortex

**Figure 4:**
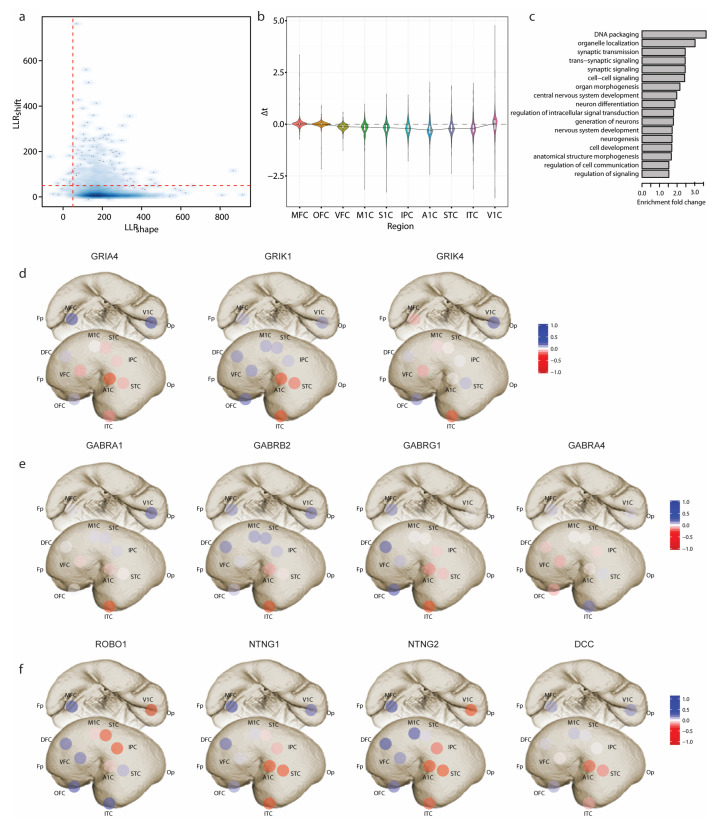
Developmental sequences in the human neocortex. (**a**) 2D density plot of ${\mathrm{LLR}}_{shape}$ and ${\mathrm{LLR}}_{shift}$. ${\mathrm{LLR}}_{shift}$ is in logarithmic scale. The cutoff is set to 50 for both ${\mathrm{LLR}}_{shift}$ and ${\mathrm{LLR}}_{shape}$ (red dashed lines). (**b**) Violin plot showing the distribution of Δt of 366 temporally-shifted genes in each neocortical area. (**c**) GO terms enriched with temporally shifted genes (*p* < 0.05). (**d**) Sequential expression of glutamate receptor genes. (**e**) Sequential expression of GABA receptor genes. (**f**) Sequential expression of axon guidance molecules. The color from red to blue shows the expression order from early to late. The Δt plotted is centered to mean. Fp—Frontal pole; DFC—Dorsolateral prefrontal cortex; OFC—Orbital prefrontal cortex; VFC—Ventrolateral prefrontal cortex; MFC—Medial prefrontal cortex; M1C—Primary motor (M1) cortex; S1C—Primary somatosensory (S1) cortex; A1C—Primary auditory (A1) cortex; ITC—Inferior temporal cortex; IPC—Posterior inferior parietal cortex; STC—Superior temporal cortex; Op—Occipital pole; V1C—Primary visual (V1) cortex.

We then applied TempShift to investigate the developmental sequences in the human neocortex. The developmental sequences represent the order in which changes in structure or function occur during the process of development of an organism. The cerebral neocortex consists of functionally distinct sensory, motor and association areas. Previous studies have suggested differential gene expression and maturation rates of different neocortical areas. To explore the developmental sequences in the human neocortex, we applied TempShift to a previously published microarray data set, including samples from 11 neocortical areas (Supplementary Table S1) across prenatal and postnatal development and adulthood [11]. Temporal dynamic genes were pre-selected by fitting a cubic regression model combining all regions (Details see Section 2). The 4153 selected genes were then used as input to TempShift. The dorsolateral prefrontal cortex (DFC) was used as the reference area (Δt=0). Since the pre-selection step is prone to select genes with the same shape, the majority of the genes (4111 genes) passed the shape selection $\Lambda_{shape}=50$. Among these genes, 366 genes were selected as multi-area temporally shifted genes with threshold $\Lambda_{shift}=50$ (Figure 4a; Supplementary Table S2). On average, the selected temporally shifted genes showed delayed development in the prefrontal cortex (MFC, OFC, DFC and VFC) (Figure 4b), inferring the hierarchical development within neocortex [16,17]. The development of the primary visual cortex (V1C), the only area located in the occipital lobe in our data, is also delayed, but with higher variation. Gene enrichment analysis showed that the temporally shifted genes are enriched in synaptic transmission and neuron differentiation (Figure 4c).

### 3.3. Sequential Expression of Neurotransmitter Receptors and Axon Guidance Molecules

**Figure A1:**
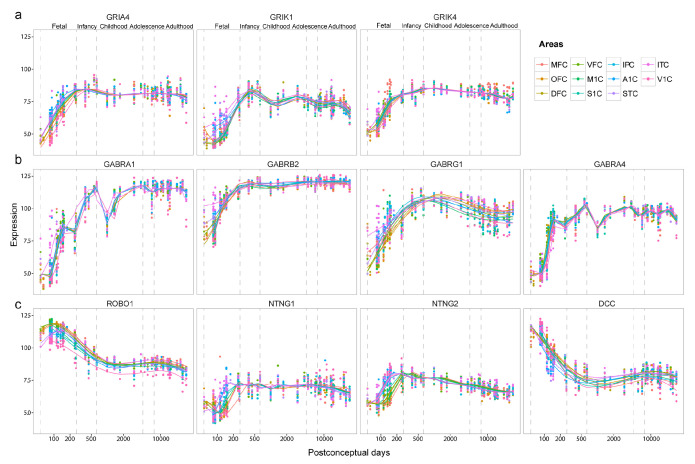
Sequential expression of selected genes. (**a**) Sequential expression of glutamate receptor genes. (**b**) Sequential expression of GABA receptor genes. (**c**) Sequential expression of axon guidance molecules.

In the synaptic transmission group, we found multiple glutamate receptor genes (GRIA4, GRIK1 and GRIK4) (Figure 4d and Figure A1a) and GABA receptor genes (GABRA1, GABRA4, GABRB2 and GABRG1) (Figure 4e and Figure A1b). GRIA4 gene encodes GluR4 subunit of AMPA receptor, which is the main subunit expressed in the late postnatal and adult. This gene demonstrated delayed expression in the prefrontal cortex and the primary visual cortex (V1C). On the other hand, GRIK1 and GRIK4 encode Kainate receptor GluR5 and KA-1 subunits, respectively. The expression of GRIK1 started from the temporal lobe (ITC, A1C, and STC), while GRIK4 started its expression sporadically from ITC, VFC and MFC to other areas. The GABA receptor genes we identified encode subunits of GABAA receptors. Among them, GABARA1, GABRB2, and GABRG1 exhibited a developmental sequence that started from ITC and then spread through the temporal lobe to other neocortical areas. In contrast, GABRA4 started from the prefrontal cortex (OFC, VFC, and DFC) and was delayed in ITC. In the neuron differentiation group, we found four genes involved in the PANTHER pathway: axon guidance mediated by Slit/Robo [12]. One way that Slit/Robo signaling mediates repulsion from the midline is by silencing the receptor of the attractive guidance cue netrin-1, netrin-2 and DCC [18]. While all four genes exhibit delayed expression in the prefrontal cortex, ROBO1 displays opposite medial-lateral temporal patterns of NTNG1, NTNT2 and DCC. ROBO1 demonstrates the medial-to-lateral pattern, with earlier expression in V1C, S1C, IPC, M1C and A1C, while NTNG1, NTNG2 and DCC demonstrate the lateral-to-medial expression (Figure 4f and Figure A1c).

### 3.4. Expression of Marker Genes Predicts the Interneuron Migration Pathway

**Figure 5:**
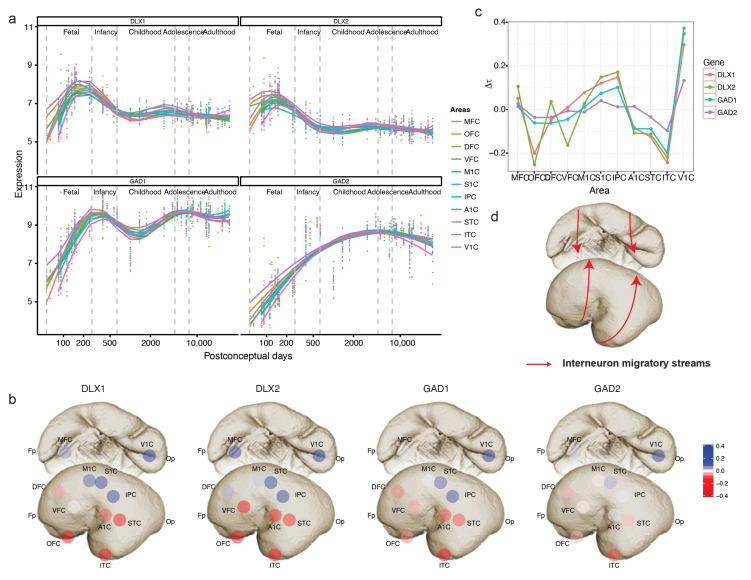
Revealing interneuron migratory streams using interneuron markers. (**a**) The expression trajectories of interneuron progenitor (DLX1, DLX2) and mature cell (GAD1, GAD2) markers. (**b**) Temporal shift of the interneuron markers in the neocortex (Δt is centered by mean). (**c**) The pattern of temporal shift is consistent across the interneuron markers. (**d**) The schematic migratory streams of cortical interneurons from ventral telencephalon.

In the above analysis, we also identified GAD1, a synthetic enzyme of interneuron neurotransmitter GABA [19], as a temporally shifted gene. The expression of GAD1, like GABA receptor genes GABRA1, GABRB2, and GABRG1, started from ITC and then spread from the temporal lobe to other neocortical areas. Unlike cortical projection neurons that derive from the dorsal telencephalon and migrate radically to the cortical plates [20], despite the existence of an additional subset of neocortex-originated neocortical GABAergic interneurons in primates, cortical interneurons mostly arise from the ventral telencephalon and migrate tangentially to the neocortex [21,22,23]. Therefore, we investigated the expression trajectories of markers of GABAergic interneurons (GAD1, GAD2) [19] and their progenitors (DLX1, DLX2) [24] to explore whether the migratory pathway of interneurons is reflected by a temporal shift in the gene expression. DLX1 and DLX2, transcription factors required for interneuron differentiation and migration [24], peaked in mid-fetal brains and were expressed until infancy (Figure 5a). On the other hand, GAD1 and GAD2, markers of pan GABA interneurons, reached highest expression in infancy following the peak of DLX1 and DLX2 expression in all neocortical areas (Figure 5a). Application of TempShift suggested that DLX1, DLX2, GAD1 and GAD2 exhibit the same temporal shift pattern of expression trajectories across neocortical areas. All four genes demonstrated sequential expression from ventrolateral to dorsomedial areas (Figure 5b,c), indicating the migratory streams of interneurons from ganglionic eminence to neocortex in humans (Figure 5d).

### 3.5. Identifying Developmental Changes in the down Syndrome

To further explore the changes in neurodevelopmental processes and related genes in DS, we applied TempShift to a published data set including complementary DS samples [13]. The original study combined this data set with the human brain development set in the previous section and only used the age-matched controls for differential expression analysis. In this section, we analyzed samples from dorsal prefrontal cortex (DFC; Supplementary Table S5) and demonstrated that TempShift is able to include all control samples by comparing gene expression trajectories inferred from age-unmatched samples and to identify DiffShape and shift genes that indicate disrupted and time-shifted biological processes.

#### 3.5.1. Increased Microglia Gene Expression in DS

**Figure 6:**
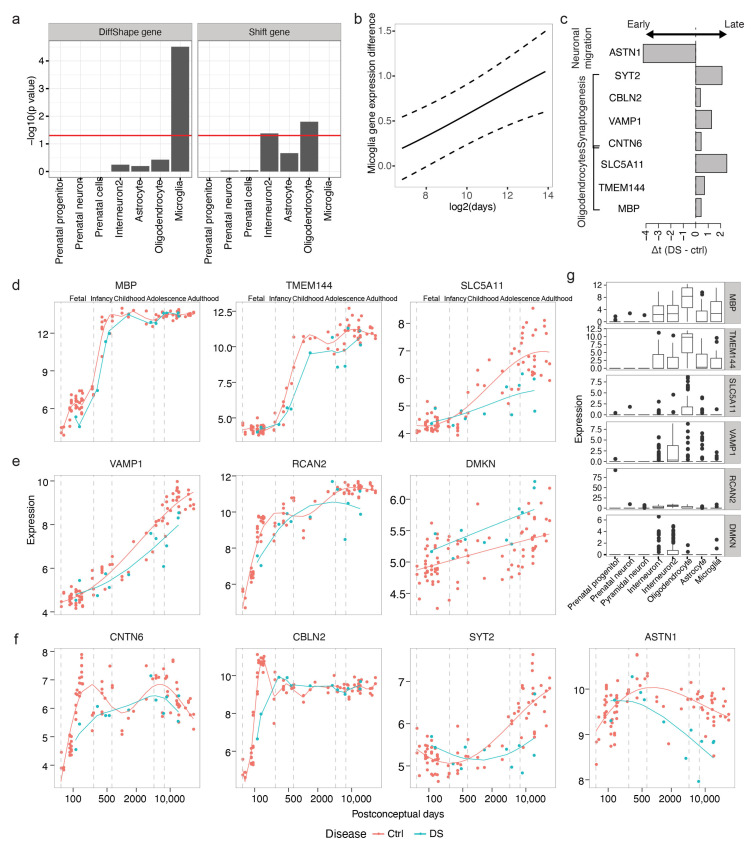
Transcriptional changes in the developmental DS brains. (**a**) Enrichment of cell type-enriched genes in DiffShape (**left**) and shift genes (**right**). (**b**) Average difference between DS and control microglia genes across development (solid). Dashed lines show standard deviation. (**c**) The time shift between DS and control of genes associated with oligodendrocyte, synaptogenesis and neuronal migration. (**d**–**f**) Expression trajectories of genes enriched in oligodendrocytes (**d**) and interneurons (**e**) and genes with known functions in neuronal development (**f**). The lines show the trajectories estimated from the shift model. (**g**) The expression pattern of genes in d and e in single cell clusters.

**Figure A2:**
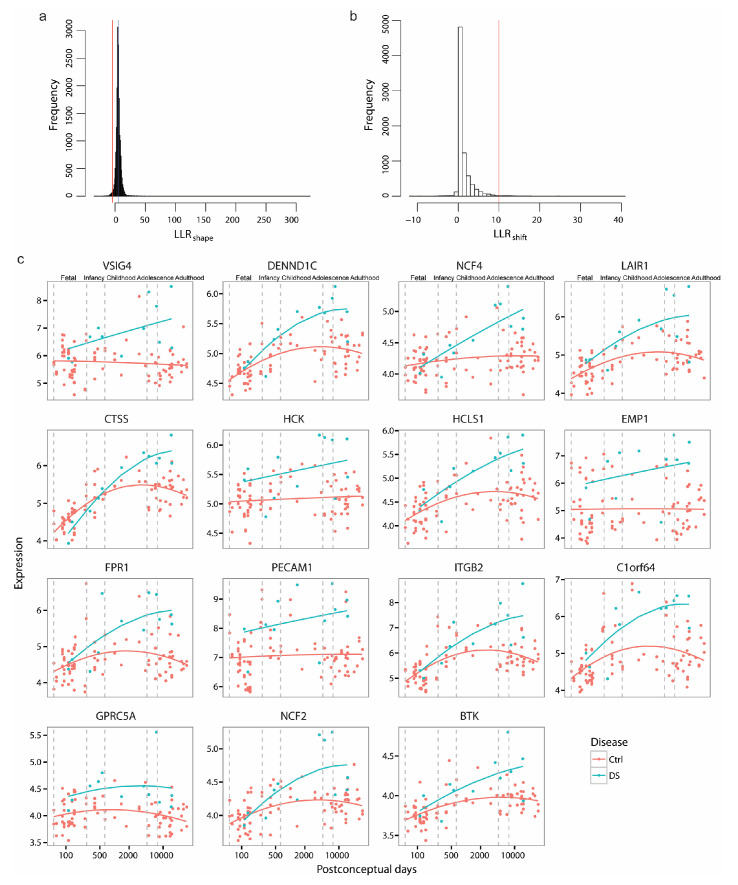
Identifying developmental changes in DS brains. (**a**) Selected DiffShape genes. (**b**) Selected shift genes. (**c**) Sequential expression of glutamate receptor genes.

We first selected DiffShape genes based on ${\mathrm{LLR}}_{shape}$ and 260 genes were selected as DiffShape genes with ${\mathrm{LLR}}_{shape}$ below two standard deviations below the mean (Figure A2a and Supplementary Table S3). By integrating with single-cell RNA-seq [14], we found that the DiffShape genes are enriched in microglia (Fisher’s exact test *p* < 0.05; Figure 6a). All 15 microglia-enriched genes show higher expression in DS than controls (Figure A2c) and this difference increases with development (Figure 6b).

#### 3.5.2. Delayed Expression of Oligodendrocyte Genes

We identified 66 shift genes with criteria ${\mathrm{LLR}}_{shape}$ above mean and ${\mathrm{LLR}}_{shift}$ greater than 10 (Figure A2b and Supplementary Table S4). Shift genes identified are enriched in oligodendrocytes (Figher’s exact test *p* < 0.05; Figure 6a). All three genes enriched in oligodendrocytes (Figure 6d) demonstrated delayed expression in DS (Figure 6c,d,g). The detected shift genes include myelin basic protein (MBP; Δt=0.43), transmembrane protein 144 (TMEM144; Δt=0.7), and solute carrier family 5 member 11 (SLC5A11; Δt=2.5). The original study selected candidate genes based on paired *t*-test and gene co-expression network analysis. Only MBP were identified by gene co-expression network analysis, while no statistics were available for quantification. None of the genes were detected as differentially expressed based on paired *t*-test combining all samples.

Another cell type found to be enriched with shift genes is interneuron 2 (Figure 6a). This cluster of interneurons is enriched with genes involved in synapses. The shift genes enriched in this cell type include vesicle-associated membrane protein 1 (VAMP1; Δt=1.3) and regulator of calcineurin 2 (RCAN2; Δt=0.4), both of which exhibited obvious delayed expression in DS (Figure 6e,g). VAMP1, also known as synaptobrevin 1, is a member of the synaptobrevin family and is involved in the docking and/or fusion of synaptic vesicles with the presynaptic membrane. RCAN2, also known as DS Candidate Region 1-Like 1 (DSCR1L1), binds to the atalytic domain of calcineurin A and has been previously associated with DS. Another shift gene enriched in interneuron 2 is DMKN; however, since the estimated expression trajectory of this gene is linear, it is indistinguishable whether this gene is upregulated or shifted leftward in DS (Figure 6e).

Furthermore, additional shift genes playing critical roles in cortical development further implied delayed development in oligodendrocytes generation, neuronal migration, neurite growth and synaptogenesis (Figure 6c,g). CNTN6, also named NB-3, encodes the neural cell adhesion molecule contactin-6 and has been found to be delayed (Δt=0.43) in expression in DS. Contactin 6 has been implicated as an autism risk gene. It interacts with NOTCH1 and promotes oligodendrocyte generation [25]. CNTN6 also interacts with cell adhesion molecule L1-like (CHL1) to regulate oriented growth of apical dendrites in the mouse neocortex [26]. Similarly, cerebellin 2 (CBLN2), a gene found to be involved in synaptogenesis induced by neurexin–neuroligin signaling [27], and synaptotagmin-2 (SYT2), a gene functioning as a Ca^2+^ sensor for fast neurotransmitter release, are both delayed in DS, suggesting delayed synaptogenesis in the DS. In addition, ASTN1 encodes astrotactin, a neuron-glial adhesion molecule that mediates glial-guided neuronal migration [27]. The expression of ASTN1 decreases earlier in the DS (Δt=−4.2), indicating a shortened neuronal migration process in the DS, which may explain the reduced brain size and altered cortical lamination in DS [28].

## 4. Conclusions

TempShift is a framework that provides flexible modeling of global temporal shift of time-series data which can deal with an arbitrary number of replicates, does not require matched time points across conditions, and can be used for two or more conditions. For ease of use, we implemented TempShift into an open-source R package (https://github.com/YingZhuLab/TempShift, accessed on 31 May 2023) in this study. At first, we validated that TempShift works well for both periodic and non-periodic data and is robust to noise through simulation. We adopted the Gaussian kernel to fit the gene expression trajectories in this study, but it can be replaced with other kernels according to the properties of the data. As we maximize likelihood using a Quasi-Newton method, which finds local maxima, we would suggest using a larger Initial value of the length scale l of the Gaussian kernel to avoid overfitting. Otherwise, multiple Initial values can be tried to get the best fit. In summary, TempShift provides a framework that can be applied broadly to study temporal differences across different conditions, such as different tissue types, disease status and species. It can be applied not only to gene expression data, but also to other time-series measurements.

The implementation of TempShift to human brain transcriptome data demonstrated the capability of TempShift in identifying shift genes and estimating temporal shift between as many as 11 neocortical areas at the same time. In addition to comparing multiple groups, Tempshift is able to detect developmental sequences of multiple biological processes in a high-throughput way. In the above application, we found that shift genes are enriched in synaptic transmission and neuron differentiation, and reconstructed the migratory streams of interneurons in the human neocortex.

Using the DS data, we demonstrated the application of TempShift to analyze groups of time-series data with unmatched ages. Using TempShift, we selected DiffShape genes and shift genes, each of which respectively suggested increased microglia, and altered regulation of developmental timing in the DS, including delayed development of oligodendrocytes, neurite outgrowth, synaptogenesis and shortened period of neuronal migration. Anatomical changes observed in DS include reduced brain size, altered cortical lamination, reduced dendritic ramifications, diminished synaptic formation, and delayed myelination [28]. TempShift successfully detected changes in these processes and provided a list of candidate genes associated with these changes in the developmental processes for future functional studies.

The TempShift detects shape difference and global shift of gene expression trajectories currently. For further studies, other models can be developed to identify more transformation of trajectories, or to refine the time interval, during which the trajectories are different in shape or temporally shifted.

In summary, we believe that not only can TempShift be used for transcriptome data analysis of the human brain, but that it also has great potential for understanding the temporal dynamics of gene expression in other biological processes.

## Data Availability

The normalized human brain microarray data used in this study are openly available in GSE25219 [11]. The microarray data of DS samples used in this study are openly available in GSE59630 [13]. The single cell RNA-seq of human neocortex used in this study are openly available in GSE67835 [14].

## Supplementary Materials

The following supporting information can be downloaded at: https://www.mdpi.com/article/10.3390/brainsci13071070/s1, Table S1: Human NCX samples.csv; Table S2: Shift genes in the human neocortex.xlsx; Table S3: DiffShape genes between DS and control.xlsx; Table S4: Shift genes in between DS and control.xlsx; Table S5: Down Syndrome samples.csv.

## Abbreviations

The following abbreviations are used in this manuscript:

OFC	orbital prefrontal cortex
Fp	frontal pole
Op	occipital pole
DFC	dorsolateral prefrontal cortex
VFC	ventrolateral prefrontal cortex
MFC	medial prefrontal cortex
M1C	primary motor (M1) cortex
S1C	primary somatosensory (S1) cortex
IPC	posterior inferior parietal cortex
A1C	primary auditory (A1) cortex
STC	superior temporal cortex
ITC	inferior temporal cortex
V1C	primary visual (V1) cortex
DS	Down syndrome

## Appendix A. The Mathematical Principle of Tempshift

In our previous study on spatiotemporal transcriptomic divergence across human and macaque brain development [6], we proposed TempShift to distinguish DiffShape genes, shift genes and no-shift genes across groups and to estimate the corresponding time difference for shift genes. This is accomplished through the following procedure. We consider one gene at a time in our analysis. Assume we have a total of m groups, and there are $n_{i}$ samples in the ith group. The total sample size of all m groups is $n=\sum_{i=0}^{m}n_{i}$. In group *i*, the time vector of length $n_{i}$ is denoted as $x_{i}$, and the corresponding gene expression vector is denoted as $y_{i}$. The combined time vector across all the samples is $x={\left(x_{1}^{T},\;x_{2}^{T},\;\ldots ,\;x_{m}^{T}\right)}^{T}$ and the combined expression vector is $y={\left(y_{1}^{T},\;y_{2}^{T},\;\ldots ,\;y_{m}^{T}\right)}^{T}$, where “T” denotes transpose. We consider the following three models: independence model (Equation (A1)), no-shift model (Equation (A2)) and shift model (Equation (A3)), where y follows a multivariate Gaussian distribution with different covariance matrices for these three models:

Independence model:

(A1) $$y\;∼\;N\left(0,\;\left[\begin{array}{ccc} {K(x}_{1},\;x_{1})\; & \cdots & 0\\ \vdots & \ddots & \vdots \\ 0 & \cdots & {K(x}_{m},\;x_{m}) \end{array}\right]\right)+\;\sigma^{2}I_{n},$$

No -shift model:

(A2) $$y\;∼\;N\left(0,\;\left[\begin{array}{ccc} {K(x}_{1},\;x_{1})\; & \cdots & {K(x}_{1},\;x_{m})\\ \vdots & \ddots & \vdots \\ {K(x}_{m},\;x_{1}) & \cdots & {K(x}_{m},\;x_{m}) \end{array}\right]\right)+\;\sigma^{2}I_{n},$$

Shift model:

(A3) $$y\;∼\;N\left(0,\;\left[\begin{array}{ccccc} K\left(x_{1},x_{1}\right) & \ldots & K\left(x_{1},x_{j}-\Delta t_{j}\vec{1_{n_{j}}}\right) & \ldots & K\left(x_{1},x_{m}-\Delta t_{m}\vec{1_{n_{m}}}\right)\\ \vdots & \vdots & \vdots & \vdots & \vdots \\ K\left(x_{i}-\Delta t_{i}\vec{1_{n_{i}}},x_{1}\right) & \ldots & K\left(x_{i}-\Delta t_{i}\vec{1_{n_{i}}},x_{j}-\Delta t_{j}\vec{1_{n_{j}}}\right) & \ldots & K\left(x_{i}-\Delta t_{i}\vec{1_{n_{i}}},x_{m}-\Delta t_{m}\vec{1_{n_{m}}}\right)\\ \vdots & \vdots & \vdots & \vdots & \vdots \\ K\left(x_{m}-\Delta t_{m}\vec{1_{n_{m}}},x_{1}\right) & \ldots & K\left(x_{m}-\Delta t_{m}\vec{1_{n_{m}}},x_{j}-\Delta t_{j}\vec{1_{n_{j}}}\right) & \ldots & K\left(x_{m}-\Delta t_{m}\vec{1_{n_{m}}},x_{m}-\Delta t_{m}\vec{1_{n_{m}}}\right) \end{array}\right]\right)\;+\;\sigma^{2}I_{n},$$

where ${K(x}_{i},\;x_{j})$ is a covariance function or kernel between groups *i* and *j*, with $n_{i}$ rows and $n_{j}$ columns. In this study, we adopted Gaussian kernel $K\left(x_{i},\;x_{j}\right)=\sigma_{f}^{2}\exp\left(-\frac{{\left(x_{i}-x_{j}\right)}^{2}}{2l^{2}}\right)$, where l denotes the length scale and $\sigma_{f}^{2}$ denotes the amplitude, which determines the average distance of the function away from its mean. Other kernel functions can be incorporated in the framework if they provide better fit to the data.

For the independence model, the between-group covariance matrix is set to a zero matrix 0 (Equation (A1)). Each position of the covariance matrix represents the correlation between the horizontal and vertical elements, and if the covariance is a diagonal matrix, then each component of the multivariate Gaussian distribution is independent of each other. For the no-shift model, all groups are combined into one group (Equation (A2)). For the shift model, the first group is set as the reference group, and for group i(i≠1), the time shift is $\Delta t_{i}$ and the shifted time vector is $x_{i}-\Delta t_{i}\vec{1_{n_{i}}}$; $\vec{1_{n_{i}}}$ is a vector of all ones of length $n_{i}$. The covariance matrix of the shift model is defined by combining the shifted time vectors into one group (Equation (A3)). The $\sigma^{2}$ is the variance of noise, and $I_{n}$ is the n×n identity matrix. Therefore, the no-shift model can be considered as a special case of the shift model when Δt=0 for all groups.

We evaluated whether a gene shows temporal shifts across groups in two steps: (1) we compared the shift model with the independence model to identify genes following the same trajectory across groups based on log-likelihood ratio ${\mathrm{LLR}}_{shape}$ (Equation (A4)); we then compared the shift model with the no-shift model to identify genes with temporal shift by calculating log-likelihood ratio ${\mathrm{LLR}}_{shift}$ (Equation (A5)).

(A4) $${LLR}_{shape}=\log\left(\frac{max\left\{L\left(\Delta t,\;\theta_{shift}|x,\;y\right)\right\}}{max\left\{L\left(\theta_{independence}|x,\;\;y\right)\right\}}\right),$$

(A5) $${LLR}_{shift}=\log\left(\frac{max\left\{L\left(\Delta t,\theta_{\mathrm{shift}}|x,\;y\;\right)\right\}}{L\left(\Delta t=0,\theta_{\mathrm{shift}}\;|x,\;y\right)}\right),$$

where Δt is the vector of time shift from the reference group, $\theta_{shift}$ and $\theta_{independence}$ are the other parameters in the shift and independence models, respectively. L(·) is the likelihood function. The parameters were estimated using optim function in R.

(A6) $$\theta_{\mathrm{independence}}=argmax\;L\left(\theta_{independence}|x,\;\;y\right),$$

(A7) $$\left(\Delta t,\;\theta_{\mathrm{shift}}\right)\;=argmax\;L\left(\Delta t,\;\theta_{shift}|x,\;y\right).$$

The likelihood function L(·) measures the likelihood that the time-series data formed by the time vector x and the corresponding gene expression vector y follow independent model or shift model. Thus, we are able to filter Diffshape genes by comparing the values of ${LLR}_{shape}$ and and select shift genes by comparing the value of ${LLR}_{shift}$.
